# Pore-Structure-Optimized CNT-Carbon Nanofibers from Starch for Rechargeable Lithium Batteries

**DOI:** 10.3390/ma9120995

**Published:** 2016-12-08

**Authors:** Yongjin Jeong, Kyuhong Lee, Kinam Kim, Sunghwan Kim

**Affiliations:** Research Reactor Fuel Development Division, Korea Atomic Energy Research Institute, Daejeon 305-353, Korea; leekh79@kaeri.re.kr (K.L.); kinamkim@kaeri.re.kr (K.K.); shkim2@kaeri.re.kr (S.K.)

**Keywords:** starch, electrospinning, nanofiber, carbon nanotube, activated carbon, mesoporous, electrochemical properties, lithium ion battery

## Abstract

Porous carbon materials are used for many electrochemical applications due to their outstanding properties. However, research on controlling the pore structure and analyzing the carbon structures is still necessary to achieve enhanced electrochemical properties. In this study, mesoporous carbon nanotube (CNT)-carbon nanofiber electrodes were developed by heat-treatment of electrospun starch with carbon nanotubes, and then applied as a binder-free electrochemical electrode for a lithium-ion battery. Using the unique lamellar structure of starch, mesoporous CNT-carbon nanofibers were prepared and their pore structures were controlled by manipulating the heat-treatment conditions. The activation process greatly increased the volume of micropores and mesopores of carbon nanofibers by etching carbons with CO_2_ gas, and the Brunauer-Emmett-Teller (BET) specific area increased to about 982.4 m^2^·g^−1^. The activated CNT-carbon nanofibers exhibited a high specific capacity (743 mAh·g^−1^) and good cycle performance (510 mAh·g^−1^ after 30 cycles) due to their larger specific surface area. This condition presents many adsorption sites of lithium ions, and higher electrical conductivity, compared with carbon nanofibers without CNT. The research suggests that by controlling the heat-treatment conditions and activation process, the pore structure of the carbon nanofibers made from starch could be tuned to provide the conditions needed for various applications.

## 1. Introduction

Carbon nanofibers are promising electrode materials for energy storage devices due to their low density, high electrical conductivity, mechanical stability, high surface area, chemical inertness, and relatively low cost [1,2,3,4]. As for application in lithium-ion batteries, carbon nanofibers need not only to provide shortened pathways for lithium ion transport, but also to offer an extensive interface with the electrolyte to promote rapid charge-transfer reactions. Therefore, a highly porous surface is needed to enhance the surface area of the carbon nanofibers. An ideal electrochemical electrode requires both a large surface area for ion storage, and an interconnected porous network with pores that are sufficiently accessible for electrolyte wetting and rapid ionic transport. Along with a large surface area, the pore structure, including size and distribution, is very important for achieving superior electrochemical properties. However, it is hard to control the pore size uniformity and shape [5].

Many researchers are developing porous carbon nanofibers using various approaches. Template-based methods have been considered the best for the synthesis of porous carbon materials with designed pore architecture and with control over the pore size distribution [6,7]. However, this requires synthesis in multiple steps, which results in a time-consuming procedure and low yield [8,9]. Among the various fabrication methods, electrospinning technology has emerged in recent years as a simple and effective approach to produce nanofibers with high surface area, from either polymer solutions or melts [10,11,12,13]. However, it is not easy to achieve precise control over the properties of the electrically conductive carbon nanofibers to produce controlled surface area or pores with narrow size distributions.

Previously, we reported a new hybrid carbon architecture consisting of CNT-reinforced carbon nanofibers with highly mesoporous structure made by electrospinning starch [14]. Mesoporous CNT-carbon nanofibers exhibit a high specific capacitance (170 F·g^−1^) and electrical conductivity (2.1 S·cm^−1^) as electrodes for supercapacitors, due to high specific surface area and sufficient pore distributions at an effective mesoporous size range of 3–5 nm. However, more research is needed on how to control the pore structure and analyze the carbon structures to be used for each electrochemical application.

Here, we report highly mesoporous carbon nanofibers reinforced with CNT, successfully fabricated using the unique lamellar structure of starch. The porosity of the CNT-carbon nanofibers was controlled by manipulating the heat-treatment conditions and the activation process. The volume of micropores and mesopores of the carbon nanofibers was greatly increased by etching disorganized carbons with CO_2_ gas. The specific area of activated carbon nanofibers was increased to about 982.4 m^2^·g^−1^. The activated CNT-carbon nanofibers show high specific capacity (743 mAh·g^−1^) and good cycle performance (510 mAh·g^−1^ after 30 cycles) due to its larger specific surface area. This provides many adsorption sites for lithium ions and higher electrical conductivity.

## 2. Materials and Methods

### 2.1. Synthesis of CNT-Starch Nanofibers

Multi-walled carbon nanotubes (MWCNTs) were chemical vapor deposition (CVD)-grown at Iljin Co. (Seoul, Korea). CNTs were refluxed in nitric acid and stirred for 5 h to attach carboxyl and hydroxyl functional groups. CNTs were then dried after rinsing with distilled water. Corn starch was used as the carbon nanofiber precursor. Prior to incorporation into the starch nanofiber films, CNT (0.02 g) was immersed in 20 mL of distilled water. Sodium dodecyl sulfate (SDS) was added to encourage homogeneous dispersion of the CNT. This solution was sonicated for 3 h in a bath-type sonicator (520 W; Hwashin Technology Co., Seoul, Korea). Polyvinyl alcohol (PVA) (0.67 g) was dissolved in 10 mL of distilled water and stirred vigorously at 80 °C. The CNT solution was then added to the PVA solution. Starch (2 g) was dissolved in 30 mL of distilled water and vigorously stirred at 50 °C. The starch solution was then boiled at 120 °C for 1 h and then cooled to room temperature. Finally, the CNT/polyvinyl alcohol (PVA) solution and starch solution were mixed and allowed to equilibrate for 1 day.

A high-voltage power supply (CPS-60K02VIT; Chungpa EMT Co., Ltd., Seoul, Korea) was used for electrospinning the nanofiber. Electrospun fiber from the CNT/starch/PVA solution was collected on an aluminum foil-wrapped metal drum with a diameter of 15 cm, rotating at 1000 r/min and held at a fixed bias of 18 kV.

### 2.2. Carbonization and Activation Process of Porous CNT-Carbon Nanofiber

The electrospun CNT-starch-PVA fiber web was stabilized by heat-treatment at 250 °C, for 1 h in air, with a ramping rate of 1 °C·min^−1^. Carbonization was then performed by heating to 700 and 1400 °C with a ramping rate of 2 °C·min^−1^ and holding for 1 h, under vacuum. After carbonization at 700 °C, the CNT/C nanofibers were activated at 800 °C for 15 min in CO_2_ (30 sccm) to increase their surface area.

### 2.3. Characterization

The nanofiber morphology was analyzed using scanning electron microscopy (SEM: XL-30S; Philips, Amsterdam, The Netherlands) and transmission electron microscopes (TEM: 20F; Tecnai, Hillsboro, OR, USA). Specific surface adsorption and pore-size distribution were characterized according to the Brunauer-Emmett-Teller equation (Tristar 3000; Micrometrics, Norcross, GA, USA). Thermograms of amylose and amylopectin from starch were analyzed by using a Thermogravimetry Analyzer (TG209F3, Netzsch, Selb, Germany). The structure of the carbon sheets in the nanofibers was characterized by X-ray diffraction (XRD: D/MAX-IIIC, 3 kW). Electrochemical measurement was performed by an automatic battery cycler system (WBCS 3000, WonATech, Seoul, Korea), using a swagelok type cell. The porous CNT-carbon nanofibers were used for the anode, as they were. Li metal foil was used as a counter electrode and the electrolyte used was 1 M LiPF_6_ dissolved in a mixture of ethylene carbonate (EC) and dimethyl carbonate (DMC) (1:1 by volume). The cells were assembled in an argon-filled glove box. Electrochemical performance was tested at a rate of 0.1 C in the voltage range of 0.01–3 V. Impedance spectroscopy was performed from 10 kHz to 10 MHz using an electrochemical analyzer (CompactStat; Ivium Technologies, Eindhoven, The Netherlands).

## 3. Results and Discussion

### 3.1. Morphology of Carbon Nanofibers

In our previous study [14], we showed that starch has advantages for fabricating a mesoporous carbon structure without using the template method. By electrospinning the starch solution, thin and flexible starch nanofiber webs were effectively fabricated. The morphology of the electrospun starch nanofibers included straight, smooth surfaces with no observable defects or beadings over the entire area of the web (Figure S1). The individual starch nanofibers were cylindrical, with a diameter of 150 nm. To maintain their fibrous morphology during the carbonization process, the electrospun starch nanofibers were stabilized at 250 °C in air for 1 h. During the stabilization process, the fibers absorb oxygen and consequently rearrange into a more stable ladder configuration. After the nanofibers were stabilized, they were carbonized by heating in vacuum (10^−6^ Torr) at temperatures from 700 to 1400 °C. Over 90% carbon content was obtained in the carbonized starch nanofibers heat-treated at >700 °C, and the carbon/oxygen ratio was increased almost linearly by the carbonization temperatures (Figure S2). This process is a demonstration of successful conversion of starch to carbon nanofibers.

**Figure 1:**
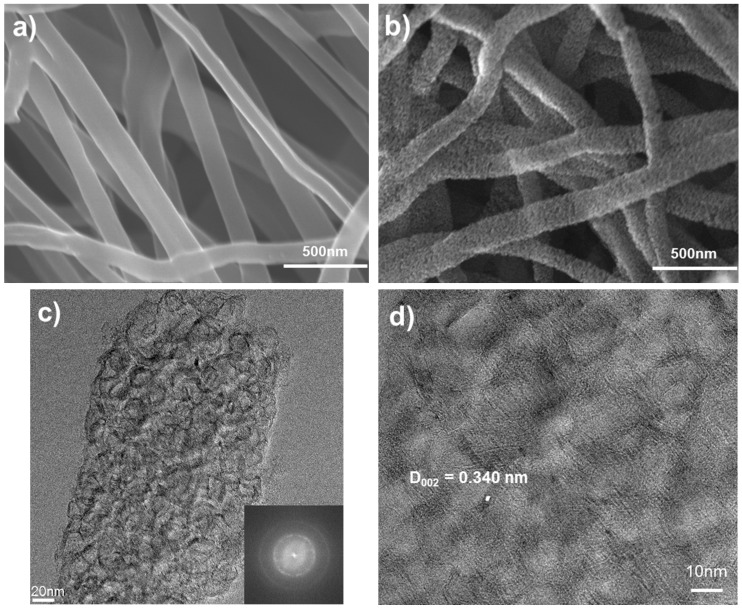
Scanning electron micrographs show (**a**) microporous carbon nanofibers carbonized at 700 °C and (**b**) highly mesoporous carbon nanofibers carbonized at 1400 °C; (**c**) transmission electron microscopy (TEM) and (**d**) high-resolution TEM (HR-TEM) images show the typical turbostratic structure created by carbonization at 1400 °C.

The surface of the carbon nanofibers that had been heat-treated at 700 °C was very smooth, as indicated by the micrographs in Figure 1a. However, the carbon nanofibers carbonized at 1400 °C exhibited a highly porous structure, as shown in Figure 1b. The transmission electron micrographs of the highly porous carbon nanofibers (Figure 1c,d) represent a turbostratic structure consisting of carbon shells of 10–15 graphitic layers around a central core, and covered by pyrolytic carbon. In general, the d_002_ spacing of graphite is 0.3354 nm, whereas the d_002_ spacing of turbostratic carbon is about 0.338–0.341 nm [15]. In a high-resolution transmission electron micrograph image (Figure 1d) the d_002_ spacing of the carbon nanofiber produced was about 0.340 nm, and this indicates that the structure of the starch nanofibers carbonized at 1400 °C was turbostratic carbon.

**Figure 2:**
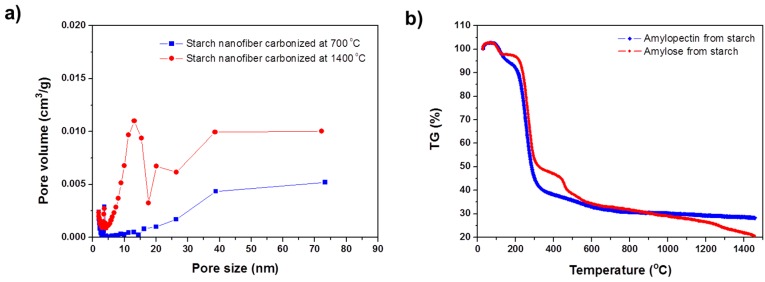
(**a**) Pore-size distributions of starch nanofibers carbonized at 700 and 1400 °C, calculated by applying density functional theory to nitrogen adsorption isotherms; and (**b**) thermograms showing the percent weight loss as a function of temperature for amylose and amylopectin from starch.

Figure 2a shows the pore size distributions obtained by applying density functional theory (DFT) to nitrogen adsorption isotherms, measured at 77 K. The starch nanofibers carbonized at 700 °C mostly contained micropores with an average diameter of 2 nm. In contrast, the pore diameters of the nanofiber carbonized at 1400 °C were distributed in the mesoporous region (5–10 nm). This novel mesoporous structure originated from the characteristic structure of starch. The starch consists of linear structures of amylose and branched structures of amylopectin. The starch microstructure shows alternating domains of amorphous amylose and crystalline amylopectin in a nanoscale lamellar structure [16,17]. Due to the difference in their crystallinity, these two polysaccharides show contrasting thermal behavior during the carbonization process. Figure 2b shows thermograms of amylose and amylopectin from the starch. A small weight loss prior to 200 °C is related to desorption of water and the drastic weight losses that occurred between 250 and 700 °C corresponded to dehydration and decarboxylation. As seen in the thermograms, amorphous amylose is almost completely decomposed at around 1400 °C and crystalline amylopectin shows more stable behavior to >1000 °C. In a native starch, the amorphous regions are located between the amylopectin crystalline layers, which are approximately 6 nm apart. Therefore, due to decomposition of the amorphous amylose regions at high temperature, the starch nanofibers carbonized at 1400 °C exhibited abundant mesopores, with an average pore diameter of 4.5 nm and the starch nanofibers carbonized at 700 °C exhibited microporous surfaces with an average pore diameter of 2.0 nm due to the pyrolysis of functional groups as seen in Table 1. In addition, though the surface area of starch nanofiber carbonized at 1400 °C was relatively lower than that of the starch nanofiber carbonized at 700 °C, the mesopore volume (*V*_me_) of it was 4 times higher than that of the starch nanofiber carbonized at 700 °C. This result suggests a new approach for tailoring both the size and the distribution of pores on the carbon nanofibers by adjusting the composition of amylose and amylopectin in the native starch.

### 3.2. Activation Process for the CNT-Carbon Nanofibers

While the carbon nanofibers show adequate pore distribution of mesopores, the surface area of the carbon nanofiber was relatively lower than that of activated carbon nanofiber. However, it is well known that the surface structure of carbon materials can be controlled by the activation process.

Moreover, hybridization with the CNT in the carbon nanofibers could be expected to result in better mechanical and functional properties. Therefore, the CNT-carbon nanofibers were fabricated using the same experimental procedure and then activated to obtain increased surface area and extensively developed internal pore structure, which make the activated carbon nanofibers highly adsorptive.

There are two main techniques for activation (physical and chemical), depending on whether gaseous or solid activating agents are used. Physically, activated carbons generally exhibit a fine pore structure, which is ideal for the adsorption of compounds from both liquid and vapor phase. The CNT-carbon nanofibers were activated physically in CO_2_. During the activation process, a reaction is triggered between carbon atoms of the carbon nanofibers and the oxidizing gas, as in the following reaction:

C + CO_2_ → 2CO (1)

This activation process etches the carbon backbones and gives rise to the creation and development of mesopores on the smooth surface of the CNT-carbon nanofibers. This is because the amorphous structure reacted faster than the crystalline structure.

**Figure 3:**
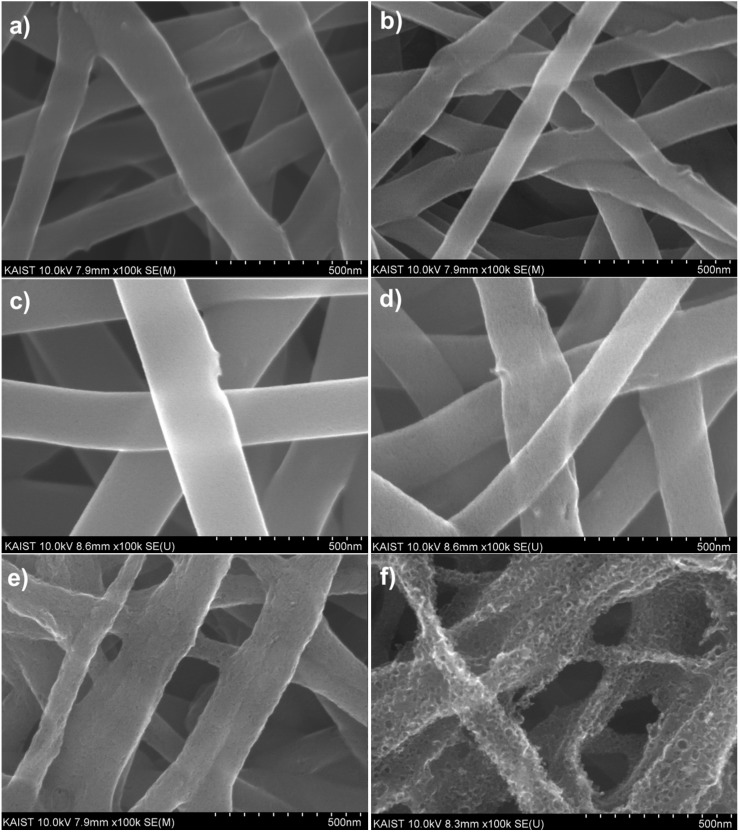
Scanning electron micrographs show (**a**) microporous carbon nanofibers carbonized at 700 °C; carbon nanofibers carbonized at 700 °C and activated at (**b**) 700 °C for 1 h; (**c**) 800 °C for 15 min; (**d**) 800 °C for 30 min; (**e**) 800 °C for 45 min; and (**f**) 800 °C for 1 h in CO_2_ gas.

The morphological changes of the CNT-carbon nanofibers were observed under the various activation conditions. Before activation, the surface morphology of CNT-carbon nanofibers carbonized at 700 °C was very smooth, as indicated in Figure 3a, and showed a mostly microporous structure. As shown in Figure 3b,c, there were no morphological changes in the CNT-carbon nanofibers under the condition of 700 °C for 1 h, and 800 °C for 15 min. However, note that the surface of CNT-carbon nanofibers becomes porous after activation at 800 °C for 30 min (Figure 3d). After this condition is achieved, the surface of the CNT-carbon nanofibers is greatly etched by the CO_2_ gas and the pore size becomes larger (Figure 3e,f).

To investigate changes in the pore structure, the specific surface area and pore size distribution of the CNT-carbon nanofibers were analyzed under the various activation conditions shown in Table 2. During the activation process, sufficient numbers of mesopores and micropores were created. As the activation time increased at 800 °C, the pore width increased from 3.1 to 6.8 nm, and the Brunauer-Emmett-Teller (BET) specific surface area decreased from 982.4 to 350.1 m^2^·g^−1^. As a result, we can conclude that the optimum conditions for activation of the CNT-carbon nanofibers should be 800 °C for 30 min. This is particularly true for the application of this material as electrochemical electrodes, because of the abundance of mesopores that provide easy access to ions and provide a higher BET specific surface area.

### 3.3. Electrochemical Characterizations of the Porous Carbon Nanofibers

**Figure 4:**
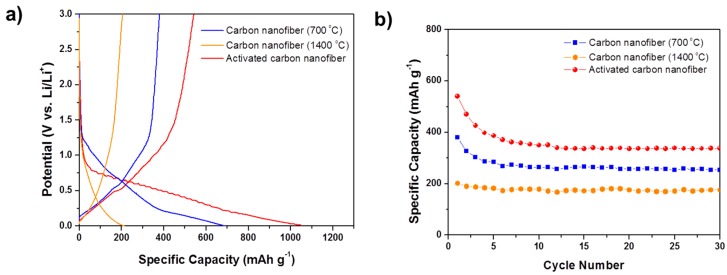
Electrochemical behaviors of the various types of carbon nanofibers: (**a**) Initial charge/discharge curves and (**b**) Cycle performance of the carbon nanofiber electrodes at a cycling rate of 0.1 C.

The electrochemical properties of the porous carbon and CNT-carbon nanofibers with various pore structures were characterized using the galvanostatic charge/discharge test. The porous nanofibers were applied for the anode as they were, because they are free-standing due to their good mechanical properties and exhibit superior electrical conductivity. Porous nanofibers with three different pore structures were used as anodes and tested at a rate of 0.1 C in the voltage range 0.01–3 V. Figure 4a shows the initial charge/discharge curves of the porous carbon nanofibers heat-treated under different conditions.

For all the types of porous carbon nanofibers, there was no significant plateau shown in the charge/discharge profiles, which is similar to that of a typical non-graphitic carbon material. Porous carbon nanofibers heat-treated at 700 and 1400 °C exhibit a reversible capacity of 380 and 202 mAh·g^−1^, respectively. The specific capacity of the porous carbon nanofiber heat-treated at 700 °C and activated at 800 °C for 30 min was enhanced to 540 mAh·g^−1^.

In the first charge and discharge cycle, an irreversible capacity loss (a common phenomenon in carbonaceous electrodes) is observed. This irreversible capacity loss arises from reduction of the electrolyte, which results in the formation of a solid electrolyte interphase (SEI) over the large surface area of the sample; or from irreversible insertion of lithium into certain positions (such as in the vicinity of residual hydrogen atoms) in the carbon material [18,19]. In Figure 4a, a gradual slope near 0.8 V appeared in the charge/discharge profiles; this represents the SEI formation. The 700 °C heat-treated nanofiber and the activated nanofiber show irreversible capacity. After these porous carbon nanofibers were charged (lithium ion inserted), a certain amount of lithium ions could not be extracted from the carbon nanofiber electrode during discharge, due to interruption by the SEI layer formed at the interface between electrode and electrolyte. From this phenomenon, we can conclude that these irreversible capacities originated from formation of an SEI layer caused by decomposition of the electrolyte, owing to the porous carbon nanofibers having relatively high specific surface area. However, the carbon nanofibers heat-treated at 1400 °C showed good reversible capacity in spite of a little lower specific capacity, which is mainly due to their turbostratic structure. The structure has partially graphitized regions and a slightly high d_002_ space of 0.34–0.35 nm (as seen in Figure 1c,d). This allows lithium ions to be easily inserted and extracted without collapsing its structure, in contrast with carbons with disordered structure, such as the carbon nanofiber heat-treated at 700 °C or activated carbon nanofiber.

The cycle performances of three kinds of carbon nanofibers are shown in Figure 4b. The charge capacity of the carbon nanofiber heat-treated at 700 °C showed some decrease over the initial few cycles, but then stabilized after six cycles and showed specific capacity of 254 mAh·g^−1^ after 30 cycles. For the activated carbon nanofiber, the specific capacity stabilized after ten cycles and exhibited a specific capacity of 338 mAh·g^−1^ after 30 cycles. The 1400 °C heat-treated carbon nanofiber showed good cycle performance, with about 90% retention after 30 cycles, due to its turbostratic structure.

The high specific capacity of the activated carbon nanofiber is derived from the enlarged specific surface area and increased adsorption of lithium ions on the internal surfaces of the nanopores formed by small graphene sheets. When graphite is fully intercalated with lithium, there is one layer of lithium per carbon layer. However, if a carbon is made up of small single layers, lithium can be adsorbed on each side of the single layers, resulting in up to two lithium atoms per six carbons, without requiring lithium atoms to occupy nearest-neighbor sites. Therefore, the carbon materials with lots of carbon sheets arranged as single layers can have high specific capacity [20]. To analyze the number of carbon sheets arranged as single layers, the empirical parameter, *R*, is used. This parameter is defined as the ratio of the height of the (002) Bragg peak to the background [21].

*R* = *B*/*A* (2)

**Figure 5:**
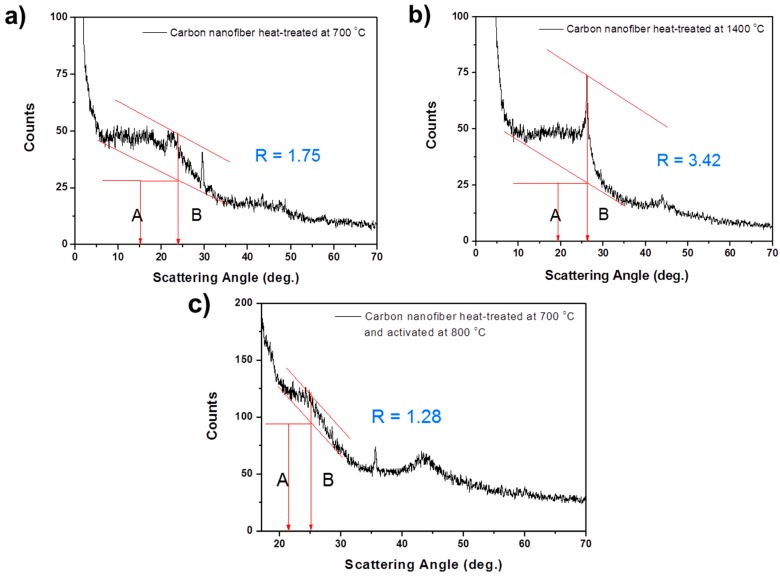
X-ray diffraction analysis for estimating the fraction of single-layers in carbon nanofibers heat-treated at (**a**) 700 °C; (**b**) 1400 °C; and (**c**) 700 °C; and activated at 800 °C.

In the XRD result, the background (*A*) is established by drawing a straight line connecting the data on either side of the peak. The peak height (*B*) is determined by drawing the line tangent to the estimated linear background, which intersects the (002) peak at a single point. To compare a rate of the single layer in the carbon nanofibers heat-treated at different conditions, XRD patterns of the carbon nanofibers heat-treated at 700 and 1400 °C, and of the activated carbon nanofibers, were obtained (see Figure 5). By calculating the R value of each kind of carbon nanofiber, we determined that carbon heat-treated at 700 °C and activated at 800 °C has the lowest *R* value (1.28). This means that it has the most single layers, when compared with carbon heat-treated at 700 °C (*R* = 1.75) and 1400 °C (*R* = 3.42). From this analysis, we can conclude that activated carbon nanofiber has a high specific capacity of 540 mAh·g^−1^ because it contains many single layers in its structure.

### 3.4. Effect of CNT on the Electrochemical Properties of the Porous CNT-Carbon Nanofibers

**Figure 6:**
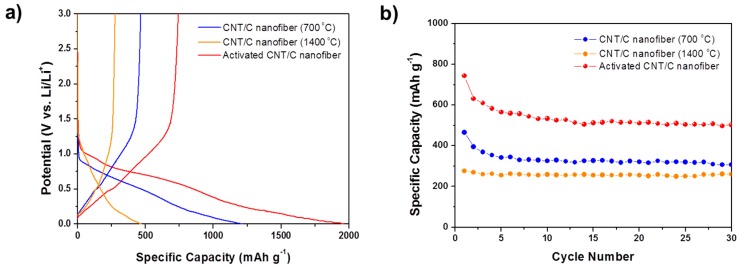
Electrochemical behaviors of the various types of CNT-carbon nanofibers: (**a**) Initial charge/discharge curves; and (**b**) Cycle performance of the 1 wt % CNT-carbon nanofibers.

Figure 6a shows the charge/discharge profiles of 1 wt % CNT-carbon nanofibers. At a rate of 0.1 C, the reversible capacity of the CNT-carbon nanofibers heat-treated at 700 and 1400 °C shows 465 mAh·g^−1^ and 276 mAh·g^−1^, respectively. The reversible capacity of the CNT-activated carbon nanofiber was shown to be very high (743 mAh·g^−1^). Irreversible capacity was also observed in the first charge/discharge profiles of the CNT-carbon nanofibers, as seen in those of the carbon nanofibers. The first discharge capacity of the CNT-carbon nanofiber heat-treated at 700 °C and activated at 800 °C, showed a very high value (1960 mAh·g^−1^), but this decreased to 743 mAh·g^−1^, less than half of the initial discharge capacity. This shows a larger irreversible capacity compared with carbon nanofiber heat-treated under the same conditions, but without CNTs. From this comparison, we know that when lithium is extracted from the CNT-carbon nanocomposite fibers (charge), it is trapped and cannot be extracted by CNTs. The plateau near 0.8 V, which is attributed to SEI formation, is also clearly visible in this sample. Therefore, we can conclude that large irreversible capacity comes from decomposition of an electrolyte resulting in the formation of the SEI layer, due to its very high specific surface area of 973 m^2^·g^−1^.

The cycle performances of the CNT-carbon nanofibers are shown in Figure 6b. For the activated carbon nanofibers, the specific capacity decreases over the initial few cycles but then stabilizes and remains at about 510 mAh·g^−1^ after 30 cycles, which is significantly higher than the theoretical specific capacity of graphite (372 mAh·g^−1^).

Compared with the carbon nanofibers, all three types of the CNT-carbon nanofibers showed enhanced specific capacity. In Table 3, specific capacities of all three types of the CNT-carbon nanofibers are summarized as a function of heat-treated conditions and their physical properties such as electrical conductivity and BET specific surface area. This high specific capacity is mainly due to its larger specific surface area, which provides many adsorption sites for lithium ions and higher electrical conductivity, compared with carbon nanofibers without CNT. Also, the CNT could effectively enhance the electrical conductivity of the carbon nanofibers due to its superior electrical properties. The electrical resistivity of the carbon nanofibers and the CNT-carbon nanofibers were measured using the 2-probe-point technique and the electrical conductivities were calculated using the following equation:

(3) σ=L/(AR)

where *R* is electrical resistance in Ω, *A* is the cross-sectional area in cm^2^, and *L* is the distance between electrodes in cm. As seen in Table 3, the CNT-carbon nanofibers showed higher electrical conductivity compared to the carbon nanofibers with the same heat-treatment and activation condition. From these results, we can see that CNT is an effective material for enhancing the electrical conductivity of the carbon nanofibers, and that it induces the increased specific capacity of the carbon nanofibers.

**Figure 7:**
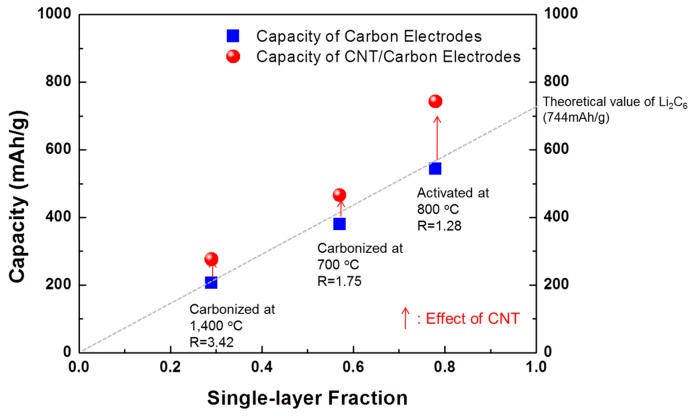
Relationship between the specific capacity and the single-layer fraction of the carbon nanofibers, and the CNT-carbon nanofibers heat-treated under different conditions.

The enhancement of the specific capacity with increase of the fraction of small graphene sheets can be predicted using a simple line plot, the capacity (0 to the theoretical value of Li_2_C_6_, 744 mAh·g^−1^) versus the single-layer fraction as shown in Figure 7. For the carbon nanofibers without CNT, the specific capacity increases with increase in the fraction of single layers in their carbon structure. These results fit well with the estimated values. However, the specific capacity of the CNT-carbon nanocomposite fibers is much greater than the value predicted value from the single-layer fraction by XRD analysis. From the result of the increase in specific capacity with the addition of CNT, we can see that CNT plays a key role in providing a large surface area (adsorbed sites) and in creating a conductive path for ions.

Therefore, the CNT-carbon nanofibers can be applied as electrodes of Li-ion batteries. Their electrochemical properties are adjustable by controlling the surface morphology and pore structure by manipulating the conditions for heat-treatment and the activation process.

## 4. Conclusions

Binder-free electrochemical electrodes of highly mesoporous carbon nanofibers reinforced with CNT were successfully fabricated using the unique lamellar structure of starch and controlling the heat-treatment conditions. Mesoporous CNT-carbon nanofibers were prepared by electrospinning starch, and in addition, we controlled the porous carbon structure through an activation process using CO_2_ gas. The electrochemical properties of these CNT-carbon nanocomposite fibers with tunable porous structure were characterized for lithium-ion battery applications. The specific capacity of 1 wt % CNT-activated carbon nanofibers had a very high value (743 mAh·g^−1^) and good cycle performance after 30 cycles, which is significantly higher than that of graphite. This high specific capacity is mainly due to its larger specific surface area, which provides many adsorption sites for lithium ions, and higher electrical conductivity, than do carbon nanofibers without CNT. From these results, we conclude that CNT plays a major role in enhancing the specific capacity of carbon nanofibers by increasing both specific surface area and electrical conductivity.

From this work, it is clear that starch has advantages (e.g., low cost and environmentally friendly) as a carbon source for electrochemical electrodes, and can provide a simple, cheap approach for the fabrication of binder-free electrochemical electrodes. Moreover, by controlling the heat-treatment conditions and activation process, the pore structure of the carbon nanofibers can be tuned to certain conditions, as needed for various applications.

## Figures and Tables

**Table 1:** Physical characteristics of the carbonized starch nanofibers. *S*_BET_: surface area; *V*_t_: total volume; *V*_mi_: micropore volume; *V*_me_: mesopore volume; *D*_A_: diameter.

Materials	*S*_BET_ (m^2^·g^−1^)	*V*_t_ (cm^3^·g^−1^)	*V*_mi_ (cm^3^·g^−1^)	*V*_me_ (cm^3^·g^−1^)	*D*_A_ (nm)
Starch nanofiber carbonized at 700 °C	451	0.23	0.20	0.03	2.0
Starch nanofiber carbonized at 1400 °C	137	0.16	0.04	0.12	4.5

**Table 2:** BET analysis of activated 1 wt % CNT-carbon nanofibers for various activation conditions.

Physical Properties	BET SSA (m^2^·g^−1^)			Average Pore Diameter (nm)	Pore Volume (cm^3^·g^−1^)		
Materials	Micro-pore Area	External Surface Area	BET Surface Area		Micro-pore Volume	Meso-pore Volume	Total Pore Volume
Before activation	458.5	21.0	479.5	1.8	0.21	0.01	0.22
Activated at 800 °C, 15 m	788.0	194.4	982.4	3.1	0.36	0.12	0.48
Activated at 800 °C, 30 m	598.8	374.2	973.0	4.1	0.27	0.26	0.53
Activated at 800 °C, 45 m	625.3	58.4	683.7	5.4	0.28	0.04	0.32
Activated at 800 °C, 60 m	224.9	120.2	345.1	6.8	0.10	0.19	0.29

**Table 3:** Electrical conductivities, BET specific surface areas and specific capacities of carbon nanofibers and the CNT-carbon nanofibers carbonized and activated at each temperature.

Heat-Treatment Conditions	CNT Addition (wt %)	Electrical Conductivity (S·cm^−1^)	BET Specific Surface Area (m^2^·g^−1^)	Specific Capacity (mAh·g^−1^)
Heat-treated at 700 °C	0	0.6	451	380
	1	1	480	466
Heat-treated at 1400 °C	0	22	137	206
	1	42	159	276
Heat-treated at 700 °C and activated at 800 °C	0	1.6	532	543
	1	2.5	973	743

## Supplementary Materials

The following are available online at www.mdpi.com/1996-1944/9/12/995/s1. Figure S1: Morphology of electrospun starch nanofibers in scanning electron micrography, Figure S2: Influence of carbonization temperature on the elemental composition of carbonized starch.
