# Supplementary material for: Pore-Structure-Optimized CNT-Carbon Nanofibers from Starch for Rechargeable Lithium Batteries
Source: Materials (Basel). 2016 Dec 8;9(12):995. doi: 10.3390/ma9120995 (PMC5457023; doi:10.3390/ma9120995)
Supplement: Supplementary file 1 [file materials-09-00995-s001.pdf]

Supplementary Materials: Pore-Structure-Optimized CNT-Carbon Nanofibers from Starch for Rechargeable Lithium Batteries

Yongjin Jeong, Kyuhong Lee, Kinam Kim and Sunghwan Kim

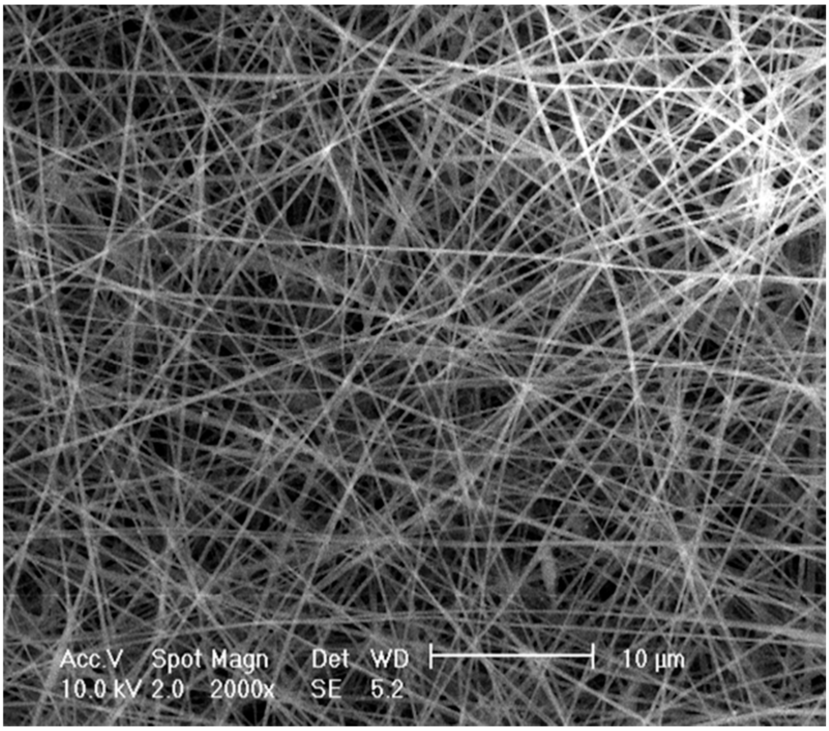

Figure S1. Morphology of electrospun starch nanofibers in scanning electron micrography.

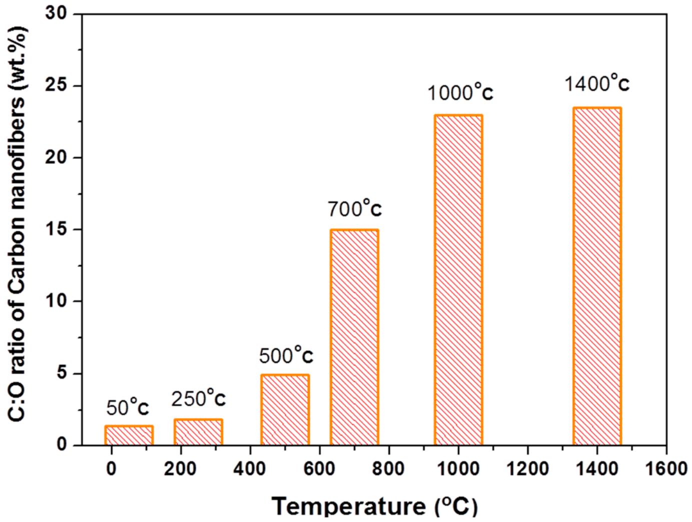

Figure S2. Influence of carbonization temperature on the elemental composition of carbonized starch.
